# Perspectives of engagement in distance debriefings

**DOI:** 10.1186/s41077-021-00192-y

**Published:** 2021-11-08

**Authors:** Cynthia J. Mosher, Alex Morton, Janice C. Palaganas

**Affiliations:** 1 Alfaisal University College of Medicine, Riyadh, Saudi Arabia; 2 MGH Institute of Health Professions, Charlestown Navy Yard, 36 1st Avenue, Boston, MA 02129-4557 USA; 3 Department of Anesthesia, Critical Care, & Pain Medicine, Harvard Medical School, Boston, MA USA

**Keywords:** Online, Distance, Debriefing, Engagement, Communities of inquiry, Remote, Healthcare simulation, Learning conversations, Pandemic, Health professions education

## Abstract

**Background:**

The COVID-19 pandemic propelled remote simulation and online distance debriefings. Like in-person debriefings, educators seek to facilitate reflective learning conversations, yet, in the online setting, educators face challenges to learner engagement that differ considerably from in-person debriefing.

**Methods:**

We performed a thematic analysis of fourteen semi-structured interviews conducted with fourteen participants who had experience with virtual debriefing as an educator or as a learner. We explored the experiences and perceptions of both educators and learners to provide a more in-depth understanding of the factors that influence engagement in online distance debriefing.

**Results:**

Our study identified the challenges online distance debriefing poses for educators and learners. We found ten themes that support the Community of Inquiry (CoI) theoretical framework and provided additional considerations related to internal and external factors of engagement, including the influence of the simulation, false engagement, and self-presence.

**Conclusions:**

We believe these findings can inform the design and facilitation of online debriefings to help provide educators with guidance and innovative solutions to best engage their learners in the challenging online environment.

## Background

The COVID-19 pandemic compelled healthcare simulation educators to respond to the suspension of in-person learning, resulting in a significant push of simulation-based learning (SBL) to online distance simulation and online distance debriefing using web conferencing platforms. Debriefing distant learners online presents considerable challenges because many aspects of debriefing are difficult to fulfil through web video conferencing [1]. The novelty of online distance debriefings is perhaps reflected through the limited information currently existing in the literature, including the identification of factors that affect engagement, which contribute to the success or failure of a debriefing.

The Community of Inquiry (CoI) framework [2], presented by Garrison et al. [3] in their seminal paper “Critical Inquiry in a Text-Based Environment: Computer Conferencing in Higher Education” provided the foundation for research in learning theory across multiple disciplines. It has been used as a framework for text-based, blended, in-person and web conferencing education over the past 20 years [4] and more recently being applied as a framework for healthcare simulation research [1, 5, 6]. We determined the CoI framework to be a best fit framework [7] for our analysis. It includes three interdependent elements: social presence, cognitive presence, and teaching presence with learners and educators assume varying degrees of all three presences as participants [8]. The CoI framework describes the dynamics of thinking and learning collaboratively online and recognizes the role of the environment and how it shapes the experience [9, 10], which is extremely important for online distance debriefing. The framework reflects much of the work now being done to guide and design the delivery of online experiential courses to support reflective learning and active engagement [9]. For this reason, we chose to apply the CoI framework to this study.

The purpose of this study is to explore the experiences and perceptions of learners and educators regarding engagement in healthcare simulation distance debriefing to provide a more in-depth understanding of the factors that influence engagement in the online setting. For the purposes of this study, we have adopted the definition of engagement from Padget et al. [11], which approaches engagement as a context-dependent state that places the responsibility for engagement upon the learning activity as well as the learner: *Learner engagement is a context-dependent state of dedicated focus towards a task wherein the learner is involved cognitively, behaviorally, and emotionally.* Specifically, we seek to answer the question of what factors influence engagement in healthcare simulation online virtual debriefing, or “distance debriefing.” Per the recommendations of the 2019 Distance Simulation Summit Proceedings, we use the term “distance debriefing” to include virtual, remote, online, synchronous, and tele-debriefings [unpublished observations/pending publication Chang TP, Elkin R, Boyle TP, Nishisaki A, Walsh B, Benary D, et al.]. In exploration of this main question, we will also determine what behaviors were observed that made participants think learners were or were not engaged and what strategies educators used that were believed to increase or decrease learner engagement.

Current literature on the topic of distance debriefing is limited, consisting mostly of studies of asynchronous distance debriefing, and focusing on the feasibility of distance debriefing [12–15]. Very little appears to be published on factors influencing engagement of learners in distance debriefing. The most recent literature points to the need for further research on distance debriefing engagement [15], the need for virtual debriefing as an essential component of virtual simulation in our current pandemic state, and the need to evaluate virtual debriefings to ensure best practices, a gap partially filled by the practical guide created by Cheng et al. [1]. These recent publications highlight the heightened interest in this topic, the need for guidance, and the concerns about engagement in virtual debriefing.

## Methods

This study used semi-structured interviews to identify the educators’ and learners’ perceptions of factors that influence engagement in healthcare simulation virtual debriefing. Within a collaborative constructivist CoI learning conversation, educators and learners assume varying degrees of teaching, social and cognitive presence as individuals [10], and the role of teacher applies to both educators and learners. For this reason, we chose to perform a thematic analysis of the data without categorizing educator or learner perspective using Braun and Clarke’s method of analysis [16].

### Interviews

The interviews were conducted using Zoom video conferencing software [17] between March and April 2021 and ranged from 25 to 45 min in duration. All interviews were recorded and transcribed by Zoom [17]. Fourteen interviews were conducted with fourteen participants who had experience with distance debriefing as an educator or learner. We used maximum variation sampling to select participants who were from different professions and geographical locations. AM, CJM, and JCP identified potential participants from personal contacts and gatekeepers, and JCP invited potential participants by email. Those who agreed to participate selected an interview date and time. AM and CJM conducted the interviews. After the first two interviews, all authors met to review the consistency of the semi-structured interview guide (see Table 1 Interview guide) and revised it for better dependability. The demographics of our participants are shown in Table 2.

**Table 1:** Interview guide

This guide reflects the general questions asked in the interviews. The interviewers slightly adjusted the wording based on whether they were interviewing a learner or educator.	
**Introduction**	
I am looking to study the factors that influence engagement in healthcare simulation virtual debriefing. My questions will be around things that demonstrate or affect engagement, including verbal, nonverbal, behavioral, and other elements that may play a role. Your responses can be positive or negative. I am more interested in your perspective as a learner/educator and what you have noticed about things that influence engagement.	
**Demographics questions**	
How many/ how often do you participate in virtual debriefings?	
How comfortable are you with virtual debriefings?	
What platform do you use for virtual debriefings?	
What learner groups/ levels do you virtually debrief/debrief with?	
How many learners are typically in a group?	
How comfortable are you with technology?	
**Interview questions**	
1. What is your definition of engagement?	
2. What did you observe that made you think learners were engaged? How did this impact engagement?	
3. When you see X, what would you speculate to be the reasons they did X?	
4. When X happened, what did that do for you? What did that do for the debriefing (conversation or learning)? E.g., one participant was nodding the entire time.	
5. What did you observe that made you think learners were not engaged? How did this impact engagement?	
6. What strategies did you use BEFORE the activity that you believe increased engagement? How did this impact engagement?	
7. What strategies did you use DURING that you believe increased engagement? How did this impact engagement?	
8. What strategies did you use BEFORE the activity that you believe may have decreased engagement? How did this impact engagement?	
9. What strategies did you use DURING the activity that you believe decreased engagement? How did this impact engagement?	
10. What cognitive aids if any did you use? How did you use it (e.g., in your head, printed or digital document, technology tools [chat box, hand raise feature, etc.]?)	
11. Is there anything else you noticed, such as nonverbal behavior or body language that you think might have influenced engagement, whether positively or negatively?	
12. Did you feel psychological safety was established and maintained during the debriefing?	
13. Did you notice anything that made you feel less comfortable that might have decreased engagement – such as visuals in the background. How did this influence engagement?	
14. What kind of debriefing training have you had?	
15. What kind of conversational training have you had?	
16. What kind of conversations-related online education training have you had?	
**Final questions**	
Our hypothesis is that there might be a correlation with comfort with technology regardless of age; however, we would like to see if age might, in fact, correlate to specific findings. This question is optional. Would you be willing to give us an age or an age range for you?	
Your geographical location	
Your cultural identity

**Table 2:** Participant demographics

ID	Gender	Age Range	Self-identified culture	Perspective	Learners	Debriefing type	Location	Profession
1	Female	> 50	White	Educator	Nursing	Distance	Newark, DE	Nurse
2	Female	> 50	White	Educator	Nursing	Distance	Toronto, Canada	Nurse Practitioner
3	Female	> 50	White	Educator	IPE	Distance	Orlando, FL	Nurse
4	Female	30-39	White	Educator	IPE	Distance	Ft Lauderdale, FL	Nurse Practitioner
5	Male	30-39	White German	Educator	Medicine	Hybrid	Birmingham, AL	Physician
6	Female	40-49	Middle Eastern Saudi Muslim	Educator	IPE	Distance	Riyadh, Saudi Arabia	Physician
7	Female	30-39	White	Learner	Medicine	Hybrid	Birmingham, AL	Medical student
8	Male	30-39	White	Learner	Medicine	Hybrid	Birmingham, AL	Medical student
9	Male	> 50	Middle Eastern Lebanese	Learner	Medicine	Distance	Riyadh, Saudi Arabia	Physician
10	Male	20-29	White	Learner	Nursing	Distance	Newark DE	Nursing student
11	Female	40-49	Middle Eastern Pakistani Muslim	Learner	IPE	Distance	Binghamton, NY	Physician
12	Female	20-29	White	Learner	IPE	Distance	Pompano Beach, FL	Occupational therapy student
13	Female	20-29	White Jewish	Learner	IPE	Distance	Brookline, MA	Genetic counseling student
14	Female	20-29	White	Learner	IPE	Distance	Somerville, MA	Occupational therapy student

Semi-structured interviews were conducted using a list of open-ended questions and prompts (see Table 1 Interview guide). The Massachusetts General Brigham IRB approved the study (ID #2020P004085, Chair O. Johnson-Akeju, January 21, 2021).

### Data analysis

The interview recordings and transcripts were uploaded to Partners DropBox [18]. Transcriptions of the interviews were created by Zoom software, and the interviewer reviewed each transcript immediately after each interview for accuracy. Any incomprehensible transcription was compared with the recording, corrected, and manually transcribed verbatim. They were deidentified; any information that might directly identify participants in the final report was excluded. The finalized transcripts were uploaded to Dedoose for coding and further analysis [19].

We conducted a thematic analysis to make sense of the shared meanings and experiences among the participants and identify the commonalities therein. Thematic analysis is a flexible method widely used in qualitative research to identify, analyze, and reveal themes within data essential to the description of the research topic under study. It entails searching across a data set to find patterns of meaning that repeat among the participant-generated data to explore explicit and implicit meanings and produce a rich and complex account of what happened and why it happened [16].

In the context of exploring engagement in distance debriefing, thematic analysis is the most appropriate method. On the one hand, it is a valuable method when investigating a topic about which little research has been conducted and exploring views of participants that are not known. On the other hand, it allows us to examine the meanings that participants attach to their and other participants’ engagement in distance debriefing and how they reflect their lived experiences and the contexts and behaviors that constrain and enable their opportunities for engagement. Furthermore, thematic analysis is not tied to a particular theoretical or epistemological position and as such it offers the theoretical freedom to provide a rich and detailed account of our data.

Prior to beginning the analysis, all authors read the transcripts repeatedly to thoroughly familiarize themselves with the data (immersion), using Dedoose to highlight and note potentially interesting information and precoding thoughts.

**Fig. 1:**
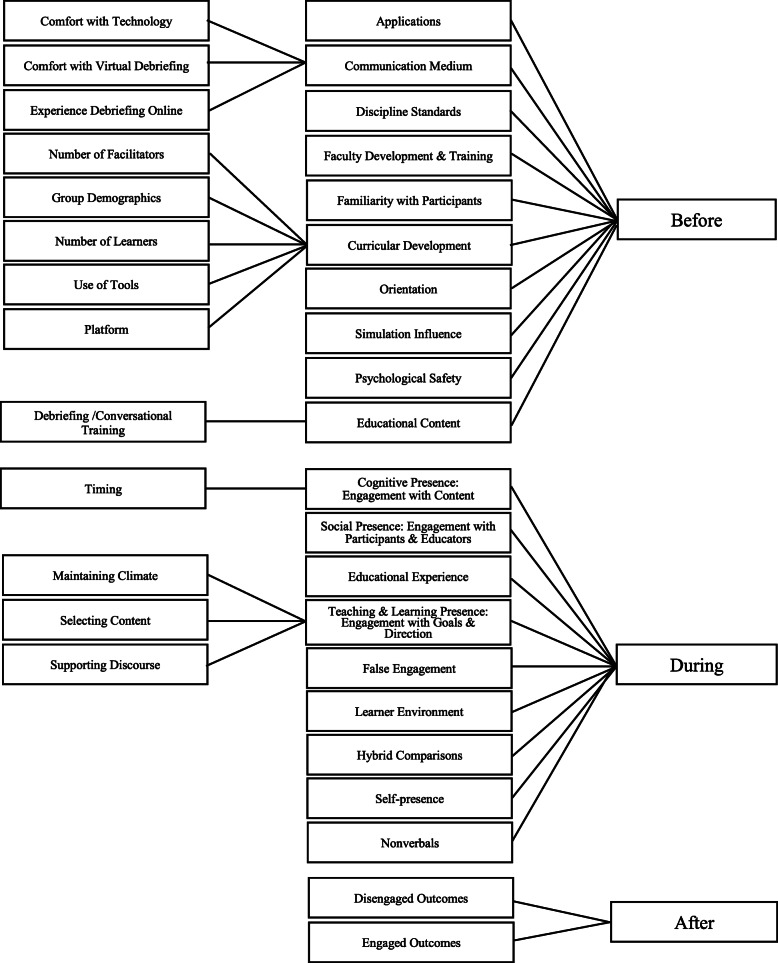
Coding tree

We created our codebook using the elements provided by the CoI framework [2]. We generated initial codes, first discussing and creating parent codes for the main elements of the framework and then child codes for the sub-elements, using the associated CoI framework element names for labeling (see Fig. 1 and Table 3). Coding was conducted a priori, remaining open to the addition of codes throughout the process. All three authors did open coding on two separate transcripts independently and met to agree on codes and code definitions.

**Table 3:** Code descriptions and examples

Code	Description	Example excerpt
**BEFORE**: That which occurs prior to the distance debriefing		
Applications	Applying a structure provided beforehand	*She used the Pearl Debriefing [Method]*
Communication Medium	Comfort with technology, comfort and experience with online debriefing	*I have become comfortable with online debriefing*
Curricular Development	Group demographics, number of facilitators, number of learners, use of tools, and platform	*Medical students.*
Discipline Standards	Standard used to design or facilitate	*Yes, it was a debriefing guide*
Educational Context	Debriefing or conversational training undertaken	*Yes, as part of my nursing course*
Faculty Development & Training	Previous training undertaken	*We did the difficult debriefing courses*
Familiarity with participants	If they were/weren’t familiar with one another prior to the debriefing	*Sometimes people would just say okay I don’t really know you let’s just go through and introduce ourselves*
Orientation	Introduction, guidelines and online etiquette	*I think it’s really helpful to set ground rules*
Simulation influence	How the simulation influenced the level of engagement	*When things were slightly more challenging or new that would encourage additional conversation.*
Psychological safety	Establishing a safe climate to engage in critical discourse	*I would say that just saying like everyone’s trying their best I know everyone wants to make this a better.*
**DURING**: That which occurs during to the distance debriefing		
Cognitive presence	Engagement with content	*More people jump in when they’re like “Oh, I felt that way too.”*
Educational experience	The contribution of the experience to learning	*It is about the technology. You’re waiting for your turn to talk.*
Hybrid comparison	Differences in a hybrid setting with learners onsite and distant	*The people that are on site in person, I feel like are always going to get that preferential treatment.*
Learner environment	Surroundings of the distant participant	*My cat will jump around me during sessions.*
Self-Presence	Preoccupation with your appearance on video	*I think that can show that you’re paying attention to what you look like in the camera.*
Social Presence	Engagement with participants & educator	*Little reactions on zoom … helps to show that you’re being listened to, somebody cares what you’re saying.*
Teaching & Learning Presence	Engagement with goals and directions	*Asking critical questions and putting you know that knowledge in a different context.*
Nonverbals	Body language, expressions and other nonverbal behaviors	*When people are leaning forward in their camera more versus like sitting, all the way back in a chair*
**AFTER**: The engagement outcomes of the debriefing		
Disengaged Outcomes	Influences causing disengagement	*If the debrief or asked like closed ended questions I feel like people didn’t engage as much.*
Engaged Outcomes	Influences creating engagement	*Being engaged encouraged engagement*
STANDALONE CODES: Additional codes to elicit needed information		
Geography	The geographic location of the participant	*Toronto, Canada*
Age	The age range of the participant	*Sure. I’m 30*
Definition of engagement	How the participant defines engagement	*I would say, like in an in-person, setting…you’re making eye contact with the group members, your face is direct. You don’t have like your head down. You’re not looking away.*

To reach consensus on coding, the authors independently coded the transcripts, which was reviewed by the other authors. Any discrepancies in coding were discussed and changes to the code book framework were made as needed. We continued to code until thematic sufficiency—no new themes could be identified from the data. Analytical memos helped us keep track of any content we found most interesting or unique. We identified 1551 code applications from 1257 excerpts. We revised the codebook and categorized them into codes under Before, During, and After, relating to engagement factors occurring before, during, or after debriefing. This resulted in a codebook of 42 codes (see Fig. 1).

We proceeded to identify themes in the data. We identified themes and subthemes, both deductively based on the CoI elements, aggregating similar codes into categories based on the CoI framework and inductively as we identified some themes outside the framework and discussed their relevance. The resulting themes were critically reviewed again and themes showing patterns of similarity or commonality were merged into a single theme, to refine and solidify our final themes. We completed cycles of coding and data theming and used the resulting themes to write the qualitative report, detailing the main factors described to influence engagement in distance debriefing.

## Results

In our analysis, a few themes were identified as most prominent, which relate to the main elements of the CoI framework [2]. Participants (see Table 2 Participant demographics) shared multiple examples of negative and positive behaviors and actions from past distance debriefing experiences that they perceived to lead to outcomes that increased or decreased engagement. Within a collaborative constructivist CoI learning conversation, educators and learners assume varying degrees of teaching, social, and cognitive presence as individuals [10] and the role of teacher applies to both educators and learners. For this reason, we chose to perform an analysis of the data without categorizing educator or learner perspective.

We found ten themes:

1. I am able to support engagement.
2. Camera on does not equal engagement and camera off does not equal disengagement.
3. False engagement is a thing.
4. Active engagement can create engagement and disengagement.
5. Tools can help but they can also hurt.
6. Body language and other nonverbals are limited.
7. Preparation, orientation, and feedback create more vibrant engagement.
8. There is a simulation effect.
9. The learner environment may be distracting.
10. Do I look okay?

### I am able to support engagement

Participants shared some very clear indicators of teaching and learning presence in the interviews. Participants acknowledged that there were strategies that exist that they could use to support engagement and also found difficulty balancing encouragement to participate without forcing the conversation:

So initially when it starts, when you don’t see students engage, you try to adjust the way you’re stating things or how you’re stating things to try to bring them along and and get them engaged. And you try to identify certain people that will respond. I try not to do that too much... So picking people (by name)... I want them to respond themselves but definitely there’s a whole thing of kind of forcing the conversation, which is really hard for me, because you don’t want to force the conversation either. (Participant 2)

You know it’s always hard because there’s a strategy that sometimes you say, ‘Okay I want to hear from everybody’ and then you say someone’s name to respond and for some students that’s fine and other ones it decreases their engagement. (Participant 2)

The feeling that calling individuals by name to participate sometimes served negatively:

If you have a disengaged learner and they’re coming from a point of view that they really don’t want to be there and they really don’t have any interest in this and they’re annoyed already, you kind of increase that level of annoyance. (Participant 5)

We found that most of the engagement factors identified were those similar to in-person debriefing, such as the use of open-ended questions, structured and “orderly debriefing,” educator passion, using the input of a learner to pose a follow-up question, interaction, active listening, and dialogue.

The selection of content to engage learners and the use of tools as venues for participation was valued by many of the participants. Polls, word gardens, emojis, and worksheets were a few of the content engagement tools felt to increase participation. Participants felt that the use of emoji showed support and encouragement. Commenting on what other learners said in discussion or in chat was mentioned as demonstrating active listening and interest in the perspectives of others and that having a lot of back-and-forth dialogue and sharing personal experiences with each other pulled others into the conversation and helped increase the feeling of cohesion and belonging. One learner described the importance of interaction with other participants:

When more individuals are engaged, I think that encourages more individuals to become engaged because … the conversation is more rich. It’s just a more fun experience to have that conversation with others. (Participant 13)

### Camera on does not equal engagement and camera off does not equal disengagement

Many participants felt everyone should have their camera on so their presence would be visual and their engagement more easily appreciated. As well, many felt having the camera off indicated disengagement and disinterest.

Not even having the camera on, not even showing your face, I think that’s something that automatically makes you feel like they’re not interested or into this. (Participant 9)

Usually if I have my video off I’m not paying attention as much. (Participant 13)

However, some felt that having the camera off does not necessarily mean they are less or not engaged.

Just because the camera is off doesn’t mean that someone’s not engaged. Sometimes they’re engaged when their cameras are off. They start speaking up and they want to debate. (Participant 3)

I’ve also had plenty of people that you know don’t want to turn the camera on or sort of in a group environment still tuning in virtually better, you know conversing a lot. (Participant 8)

I think that’s just like a perception thing that if you’re not if I can’t see your face I don’t think you’re paying attention. (Participant 13)

Other indications of visual presence and engagement absence were described as well:

Repeating questions, and even the length of their responses. Sometimes if it’s just a really short flippant response you realize they’re not as engaged in the experience. (Participant 2)

Cameras off led some participants to do the same, described by one as conveying a sort of permission.

If I’m the only one, or one of the few people that have their camera on… it makes it, I don’t know if it’s a less comfortable sort of thing but it’s just sort of like, well if nobody else is doing it…why am I doing this? (Participant 8)

One unique description of camera off not being an indicator of disengagement but rather a cultural influence was shared by an educator:

Sometimes people, especially here for females they prefer to close the camera… you know it’s easier for them so they (don’t have to wear) hijab (head/face covering). (Participant 6)

### False engagement is a thing

Sometimes cameras are on but the participants are not really there, which was described by one educator as “false engagement”:

They’re like doing something else and they’re just they're on camera just to be there. (Participant 3)

Other instances of false engagement included:

They weren’t engaged. They were just looking for the answers in the assignment...They’re like doing something else and they’re just they're on camera just to be there... (Participant 14)

False engagement was felt to be significant because behaviors that would normally indicate engagement may actually be quite the opposite.

I can bobble (nod) my head but I might not be fully paying attention. (Participant 14)

### Active engagement can create engagement and disengagement

It (the active engagement of others) improves my learning. It improves my engagement and my drive to learn more. (Participant 5)

Active engagement being a catalyst for creating engagement as well as causing disengagement is not specific to distance debriefings. It occurs in face-to-face debriefings as well. We will examine how management of these issues differ in the distance setting in the discussion.

While active engagement was felt to be a general driver of group engagement, a few individuals dominating the discussion can have a significant impact on the engagement of others, causing those quiet to feel they don’t need to participate and causing those who want to participate to feel silenced.

There were a few people who kind of took the lead and it made it so that way not everyone's voice can be heard. (Participant 4)

I’ve seen decreased engagement when you have someone that responds to all the questions. (Participant 2)

One interesting outcome of this juxtaposition of engaged and disengaged participants was increased engagement for those already engaged, though this was not always expressed in a positive light.

I felt like I had to speak more because other people were not engaged. (Participant 7)

The active engagement of the educator was said to play a positive and negative role. An excess of interjection by the educator was felt to disrupt the flow of discussion, leading to disengagement of learners, while a few described the outcome of an individual’s active engagement as serving to increase interaction of the educator with them.

Facilitators usually call on those who speak more. (Participant 12)

You can feel it and sense it so as a facilitator, I get more engaged… it’s kind of something that’s contagious when people are engaged. (Participant 2)

Turn-taking was an approach used by some educators to try to encourage participation and engagement but it was actually felt to contribute to disengaged outcomes.

If you have an idea, you want to participate and other people are talking and so on, so that when you get your turn probably the idea is out of context of the subject matter that's under discussion at that time. (Participant 9)

### Tools can help but they can also hurt

Several tools of the web conferencing platform were said to be useful for engagement, including screenshare, whiteboard, handraise, polls, and chat. Most tools were used minimally and found to be helpful; however, use of chat was the most controversial, with both positive and negative contributions to engagement. Some learners felt it contributed to disengaged outcomes, such as people paying more attention to chat than to the person speaking, when it becomes a side conversation between participants, that discussion in chat has no flow, that it is distracting, and that the side conversations in chat can be missed or fall out of relevance because the discussion has proceeded to another topic. Nonetheless, chat was found by some to be of benefit in small doses and particular uses and for some was the engagement tool of choice.

Saying ‘Oh, I felt the same way’ that’s a perfect opportunity for me to just write in the chat box and say, yes, I felt the same way, without interrupting what they’re saying, but still participating in the conversation. (Participant 13)

It (chat) was used more than video. People are more comfortable typing in responses than turning on their video. It’s better than no engagement. (Participant 7)

Watching for the unmute action of participants was mentioned as an important alert to someone’s intent to contribute to the discussion.

Just watching when people unmute I think that that’s something that I’ve like learned to get really good at to tell if anybody else is going to talk, if you have like the screen of people who are all muted, you can see when somebody unmute and then you know that they’re going to say something so just being aware of that. (Participant 13)

### Body language and other nonverbals are limited

The difficulty of perceiving body language and nonverbal indicators of engagement was expressed by many of the participants. Some of the limitations shared were the inability to see all participants at once, the camera only showing individuals from the neck up, and the need to stay in the frame of the camera, which limits your movement. One suggestion from a learner was to use portrait mode so that you can actually see the hands and more of the body language. Eye contact was also said to be difficult to perceive given camera and display angles, but when present could also be a very strong indicator of engagement.

Almost all the students were sitting forward, their eyes are open… they had these nonverbal expressions of true engagement. (Participant 2)

Facial expressions and nodding of the head were prominent nonverbal indicators of engagement while having your head down as if you are engaged with something else and leaning back in your chair demonstrated a lack of engagement.

You can tell when people are leaning forward in their camera more versus like sitting, all the way back in a chair, so I think a lot of it is based on like body motion. A lot of head nodding, I think that’s a huge one, since most of us sit on mute until it’s our turn to talk. (Participant 13)

### Preparation, orientation, and feedback create more vibrant engagement

Some educators found that setting a friendly tone in some sort of pre-simulation activity and getting to know one another proved to be very helpful. Examples included using an ice breaker:

It’s like some sort of like ice breaking some sort of activity, like two three minutes long... For example, ‘What is a good thing that happened today or this week that you want to share.’ or like ‘Do you want to tell us what’s outside your window?’ Those small things that would bring us back together and because it’s all in the sense of being in a team, working together over the Zoom and learning together is the thing that we forget when we are in virtual environment. (Participant 5)

Unfamiliarity with other participants was felt to be a factor that could make a debriefing uncomfortable, making the debrief itself more difficult to engage. Helping to develop a familiarity with one another by calling participants by their name, addressing the person who spoke before you, was conveyed as ways to get to know one another at the start of the debrief.

I know early on, sometimes people would just say okay I don’t really know you let’s just go through and introduce ourselves and even something as simple as that, at least gets people talking. (Participant 8)

When they knew what the discussion is going to be around…they have an idea, and they have questions. So when we start the discussion or the debriefing they will be more engaged. (Participant 6)

A more informative prebrief was identified by one educator as key to increasing engagement:

What I perceive and what we got in evaluations was we needed to do a more sophisticated prebrief. And when we did the more sophisticated pre briefing that we got more interaction...just to kind of give them that warning shot that this is a very different style of simulation than what they're used to and also just being very specific about the flow. (Participant 1)

### There is a simulation effect

Interestingly, the interviews described the influence of the simulation itself and how it contributes to engagement. One learner stated very clearly that if the simulation activity was of interest to her, she was more active in the debriefing conversation, and if it was dull and boring or was a typical case she felt unmotivated in the discussion. As we explored this more with participants, we found they considered it to be a prominent factor of engagement in the debriefing conversation.

Whatever happens in the simulation automatically influences the debriefing because it influences the dynamics of the group that you’re debriefing. (Participant 14)

I think we did have a discussion of, like, this is why you get a full history, even in those times when people are trying to rush you. So oh yeah I think that ends up being a pretty good discussion with that sort of twist there. Sometimes you can bring in certain discussions of maybe ethics or… what’s the patient outcome after this intervention that you’re doing right now. So there’s a lot of ways to kind of spark discussion. (Participant 8)

Interviews described how learners found value in seeing how their peers interacted with the same patient and this was found to increase their desire to engage in the debriefing conversation:

They got to see different techniques and that this was extremely valuable for their engagement in the debriefing. (Participant 1)

### The learner environment may be distracting

The environment surrounding the learner at their distant location presents challenges unique to debriefing online. Distractions that diverted attention included seeing background visuals, participants laying on their bed or moving around in their space, a distracting virtual background, a “baby in their lap,” and noise interruptions.

Learners using earphones as a tool to lockout surrounding distractions and focus on the debriefing conversation was seen to be a strong sign of engagement.

Mentions of environment also included descriptions of hybrid simulations where a group of students were onsite and a group of students in their individual homes or workplaces. Generally, hybrid settings for debriefing was felt to create feelings of disconnect and exclusion:

The people that are on site in person, I feel like are always going to get that preferential treatment. You know you just have sort of the Zoom on a screen, but the people that are in person, are all sort of facing each other and let’s say the attending you know even has their back to the screen or not really looking... that sort of shows like okay well clearly there’s this sort of preference to (those) in-person… I’ve been the one in person, so I can kind of see like okay whoever’s there virtual is going to have a very difficult time sort of joining in when again they don’t sort of take time to you know, bring in the people that are virtual. (Participant 8)

The reverberating effect of this perception was clearly stated:

I think the fact that we are splitting it and doing hybrid I think is a factor of disengagement. (Participant 5)

### Do I look okay?

The online learning environment creates a unique “self-presence” not captured in the CoI framework. We found a theme of self-consciousness, a heightened sense of awareness with one’s own personal appearance and presence in the video screen. This presence was identified by several of our participants and aptly described by one:

I think sometimes people, when you’re talking to them, they will be, like doing this and doing that [motioning to show adjusting their appearance]. So yes, I think sometimes people will be disengaged when they are looking towards the camera. They’re not looking at other people to see them... they are looking at themselves. (Participant 6)

## Discussion

Many best practices of in-person debriefing were present in the interviews, indicating that much of what we do in-person is transferable to the online environment. Nonetheless, the factors identified by our participants demonstrate the unique challenges faced in distance debriefing engagement. We discuss here a select few that we found to be the most prominent and interesting as well as their implications in practice.

### Promoting and balancing participation

Like in-person debriefing, promoting and balancing participation were noted by participants as important to engagement. The limitations of distance debriefing, as well as the higher ease of disengagement innate to online platforms, may amplify the educator’s perceived need to participate more in discussion to encourage engagement. Garrison [9] attributes the level of teaching presence with a correlating rise or fall in learner social presence, saying that an educator who models a strong social presence will raise the social presence of learners. Our findings suggested that there is a potential for the opposite effect.

Frequent interjection by the educator can cause a disruption in the discussion and reduce engagement of participants. Having a moderator or co-debriefer to privately signal the educator who is interjecting too frequently can be a helpful strategy. To aid the educator in promoting engagement of participants, the moderator or co-debriefer can manage the use of web conferencing platform tools such as posting a question for replies, presenting a poll for voting, and promoting handraise for verbal participation.

One or more individuals dominating the discussion, which may be unintentionally encouraged by an educator eager for participation, can also dampen engagement. This may occur due to lack of participation causing the vocal few to feel obliged to fill the gap or can arise from a very gregarious learner. To set the stage for balanced participation, the educator should conduct an orientation that introduces paths for engagement through the use of platform tools and using and encouraging such engagement options frequently throughout the debriefing. Additionally, the educator can use techniques applied in the in-person debriefing setting such as redirecting the input of those actively engaged to create opportunities for others [20], which can be done verbally or through the use of a platform engagement tool such as a poll.

Familiarity with participants was a topic that came up frequently as many felt it to be an important factor for everyone to engage in discussion more comfortably. Establishing this familiarity by way of brief profiles shared in advance or by having an introduction or ice breaker activity as part of the orientation may help participants feel more comfortable to reflect on their experiences and explore their knowledge gaps with the group.

The in-person setting is conducive to establishing a warm and welcoming atmosphere because the presence of everyone in the room is “real” and psychological safety is more readily established and maintained. In the distance setting, participants need to feel acknowledged as a “real” person behind the screen. Establishing and maintaining this feeling is essential to promoting engagement. The educator can engage in “transparent participation” by describing their own perceived difficulty of engagement when using online platforms to normalize such feelings shared by participants.

To help establish a more in-person feeling the educator should initiate the debriefing with a friendly tone, injecting humor where suitable, calling individuals by their names, and giving direct and prompt feedback. Establishing and maintaining a psychologically safe environment [21] in a distance debriefing is challenging due to external factors beyond the educator’s control, such as background visuals and random passersby within video camera view. This can be addressed by emphasizing to participants the need for privacy, suggesting the use of a non-distracting virtual background, and encouraging the use of headphones or other audio devices to reduce background audio sounds and prevent anyone in the participant’s area from hearing the debriefing discussion.

The use of chat as a means to participate in the discussion is a point of controversy. Participants reported the positive use of chat to increase levels of engagement and facilitate it more readily for individuals who are shy or hesitant to speak up in an online distance conversation or within a larger group of participants. Nonetheless, due to the nature of chat as somewhat of a side conversation, excessive use can increase cognitive load and become a distraction that may compromise overall engagement. Having a co-debriefer or moderator to monitor and direct chat allows the educator to focus on the active debriefing discussion. If a co-debriefer is unavailable, the educator can assign a learner to assist in monitoring. Optionally, the educator can transparently inform the group when they followed the chat and when they were unable to do so and invite any points made in chat to come forward.

Balancing participation in a simultaneously hybrid in-person and distance debriefing requires more thought and planning as well as inclusive and intentional facilitation by the educator. Orientation to the hybrid process and invitation to participate “as if in-person” can establish paths for engagement. Positioning of audio-visual equipment that allows a full view of the participants onsite with good quality audio will be important so that online participants can hear and see clearly. Positioning of monitor and seating, as well as directed facilitation during the conversation within topics (versus at the end of each topic) may elicit and sustain debriefing engagement.

### The visuality of engagement

Being physically present facilitates many aspects of interaction that are easily seen, such as body language, eye contact, and facial expressions. In an online web conferencing setting, visuals can be more difficult to appreciate and sometimes impossible to discern due to participants having their camera focused only on their face, having multiple screens with the camera on the screen that they are not facing, dark or very bright lighting, or having their cameras off. Based on our participants’ perceptions, we understand that audio without camera can create active engagement; however, being able to see one another provides a visual that brings the experience closer to feeling like that of being in-person and can allow others to perceive engagement through the visual it offers. While having the camera on does not ensure a person is engaged in the activity, having the camera off completely removes the possibility of discerning any nonverbal engagement.

Multiple strategies can be used to improve the visual benefits of having audio and video on. Providing guidelines for participation that favors good audio and video will help eliminate disturbances in attention. The guidelines should accommodate or provide options for learner preferences. What guidelines are most appropriate and how to best balance educator preferences with learner preferences is beyond the scope of this article and is an area for future studies.

Another important aspect regarding visuality of engagement is the number of learners. Platforms have limitations in gallery view wherein one can view all participants on one screen and should be considered in planning. The Zoom platform standard gallery view accommodates 25 participants, though settings can be expanded to increase this number. However, increasing the number of participants decreases the ability to perceive nonverbal communication and body language, which some raised in discussion as important to creating a more in-person feeling in the online setting. The ideal number of participants in distance debriefing is a topic of research to be explored.

Particular attention to unmute indicators provides signaling for engagement and participation. When a learner places themselves on mute to avoid causing audio disturbance then unmutes during a conversation, it may be a sign that they wish to speak. This should be quickly picked up on by the educator so that person is invited to the ongoing conversation.

A test-run of camera settings, including positioning, lighting, and background may help decrease the distractions of “self-presencing”. Disabling the view of oneself is a setting option available in Zoom [17] and most platforms and may eliminate the distraction of self-presence for some but may paradoxically increase the cognitive load that can occur for others when they can’t see themselves. However, enabling and enlarging the view of oneself during the conversation may also provide assurance, depending on the individual. This, too, is an area for future research. Trialing all suggestions here to determine which works best for one’s self-presence may assist in decreasing distractions in future debriefings.

### The simulation as a mediator of engagement

In his book, “E-learning in the 21st century: A framework for research and practice, 3^rd^ edition,” Garrison [9] states that cognitive presence, one of the three main presence categories in the CoI, is operationalized by the Practical Inquiry model, the first phase of which is a “triggering event.” He describes it as the instigator of cognitive presence, specifically as “a well thought-out activity to ensure engagement and buy-in from the group.” In general, online instruction, this refers to the learning activity that the educator creates and presents to learners *during* the activity as the “trigger” for their learning conversation. However, this “triggering event” in healthcare simulation debriefing occurs *before* the learning conversation. It is the simulation itself. As stated repeatedly by our participants, a scenario that is not typical, that brings in something unexpected, controversial, or new will heighten the interest of the participants and make them eager to discuss what they observed. The emotions during a simulation experience “rolls into” the debriefing and may range from boring to challenging to traumatic, acting as a mediator to debriefing engagement. Viewing a simulation online is an experience different from in-person experiences. Critical to hybrid simulations, it is important to consider designing a simulated case to make the engagement opportunities as equitable as possible for both in-person and distance participants.

## Strengths and limitations

A key strength of this study was that we interviewed stakeholders from three different countries and diversified our participants as much as possible by way of profession, group demographics, and learner/educator perspectives. The findings of this study are limited to the lived experiences of the 14 participants. Despite the diversity of our participants, we did not specifically study the difference in culture or profession and may have underestimated them.

## Future research

Our findings reveal that distance debriefing presents different challenges that call for new standards and guidelines unique to online experiential distance facilitation—a needed area for future research.

We initiated our research using the CoI Framework by Garrison [2] and found additional codes in our first and continued rounds of coding. The elements in distance debriefing that are not specifically addressed in the CoI framework and would be worthy of in-depth research include:

- Self-presence
- False engagement
- The role of the simulation in promoting engagement
- The interplay of internal and external factors of engagement
- The time sequential process of each element
- Cultural factors of engagement

There is more research on this topic amongst different cultures and socioeconomic groups. Internet connectivity as well as web conferencing technology may be resources of privilege which impact participation and may impact engagement for those new to technology or in areas where connectivity is lacking.

Participants choose options that best fit their comfort level—comfort that is essential to supporting engagement. How to best balance the engagement needs of the educator with the engagement needs of the learner is in need of future research. Some educators provide strict guidelines, while others do not provide any guidelines. Understanding how to create guidelines and an orientation that sets up the best possible distance debriefings while still promoting a natural conversation is needed. As well, the ideal number of participants in a distance debriefing is an important topic for investigation.

## Conclusions

This study describes the experiences of participants in distance debriefing to shed light on the influences of engagement, some of which occur in in-person debriefing and present in unique and challenging ways in the online environment. We found the CoI framework to be a flexible but rigorous framework that proved a good ‘fit’ for our study. We also found additional elements beyond the CoI framework that appear in simulation-based distance education that can inform future distance simulation research using the CoI framework.

Current best practice simulation standards [22] do not address specific problems faced by learners and educators in online distance debriefings. The findings from our interviews provide additional considerations that may aid the development of new standards specific to online distance debriefing.

## Data Availability

The dataset is not available, per approved IRB.

## Abbreviations

CoI: Communities of Inquiry
COVID-19: Coronavirus disease of 2019
INACSL: The International Nursing Association for Clinical Simulations and Learning
IRB: Internal Review Board
CJM: Cynthia Mosher
AM: Alex Morton
JCP: Janice C Palaganas
SBL: Simulation-based learning

## References

[1] Cheng A, Kolbe M, Grant V, Eller S, Hales R, Symon B, et al. A practical guide to virtual debriefings: communities of inquiry perspective. Adv Simul. 2020. 10.1186/s41077-020-00141-1. 10.1186/s41077-020-00141-1 PMC7422458 32817805

[2] Garrison D, Akyol Z, Moore M (2013). The community of inquiry theoretical framework. Introducing the 2013 handbook of distance education.

[3] Garrison DR, Anderson T, Archer W (2000). Critical inquiry in a text-based environment: computer conferencing in higher education model. Internet High Educ.

[4] Castellanos-Reyes D (2000). 20 Years of the Community of Inquiry Framework. Tech Trends.

[5] Cooper VA, Forino G, Kanjanabootra S, von Meding J (2000). Leveraging the community of inquiry framework to support web-based simulations in disaster studies. Internet High Educ.

[6] Liu W, Wang J, Zhang H, Yu C, Liu S, Zhang C, et al. Determining the effects of blended learning using the community of inquiry on nursing students’ learning gains in sudden patient deterioration module. Nursing Open. 2021. 10.1002/nop2.914. 10.1002/nop2.914 PMC8510760 33973718

[7] Carroll C, Booth A, Leaviss J, Rick J. “Best fit” framework synthesis: Refining the method. BMC Med Res Methodol. 2013. 10.1186/1471-2288-13-37. 10.1186/1471-2288-13-37 PMC3618126 23497061

[8] Garrison DR, Rogers PL, Berg GA, Boettcher JV, Howard C, Justice L, Schenk KD (2009). Communities of inquiry in online learning.

[9] Garrison DR. E-learning in the 21st century: a framework for research and practice. 3rd ed: Routledge; 2017.

[10] Gordon R. Debriefing virtual simulation using an online conferencing platform: lessons learned. Clin Simul Nur. 2017. 10.1016/j.ecns.2017.08.003.

[11] Padgett J, Cristancho S, Lingard L, Cherry R, Haji F (2019). Engagement: what is it good for? The role of learner engagement in healthcare simulation contexts. Adv Health Sci Educ Theory Pract.

[12] Verkuyl M, Atack L, McCulloch T, Liu L, Betts L, Lapum J, et al. Comparison of debriefing methods after a virtual simulation: an experiment. Clin Simul Nur. 2018. 10.1016/j.ecns.2018.03.002.

[13] Verkuyl M, Lapum J, Hughes M, McCulloch T, Liu L, Mastrilli P, et al. Virtual gaming simulation: exploring self-debriefing, virtual debriefing, and in-person debriefing. Clin Simul Nur. 2018. 10.1016/j.ecns.2018.04.006.

[14] Miller E, Farra S, Simon A. Asynchronous online debriefing with health care workers: Lessons learned. Clin Simul Nur. 2018. 10.1016/j.ecns.2018.04.007.

[15] Bradley CS, Johnson BK, Dreifuerst KT. Debriefing: a place for enthusiastic teaching and learning at a distance. Clin Simul Nur. 2020. 10.1016/j.ecns.2020.04.001. 10.1016/j.ecns.2020.04.001 PMC7218380 32406399

[16] Braun V, Clarke V, Cooper H (2012). Thematic analysis. APA handbook of research methods in psychology. Vol. 2, Research designs.

[17] Zoom (5.4.7.). San Jose: Zoom Video Communications, Inc. 2021

[18] Dropbox (122.4.4867). 2021. San Francisco: Dropbox, Inc. 2021

[19] Dedoose (8.3.47). Los Angeles: Sociocultural Research Consultants, LLC. 2021

[20] Grant VJ, Robinson T, Catena H, Eppich W, Cheng A. Difficult debriefing situations: a toolbox for simulation educators. Med Teach. 2018. 10.1080/0142159X.2018.1468558. 10.1080/0142159X.2018.1468558 29792100

[21] Edmondson A (1999). Psychological safety and learning behavior in work teams. Adm Sci Q.

[22] INACSL Standards of Best Practice. Simulation^SM^ Debriefing. Clin Simul Nur. 2016. 10.1016/j.ecns.2016.09.008.

